# Supplementary material for: The Dorsomedial Prefrontal Cortex Uses Reward Predictions to Regulate How Rewards Are Pursued
Source: bioRxiv. 2026 Sep 3:2026.08.29.748037. Preprint. [Version 1] doi: 10.64898/2026.08.29.748037 (PMC13684574; doi:10.64898/2026.08.29.748037)
Supplement: Supplementary file 1 [file media-1.pdf]

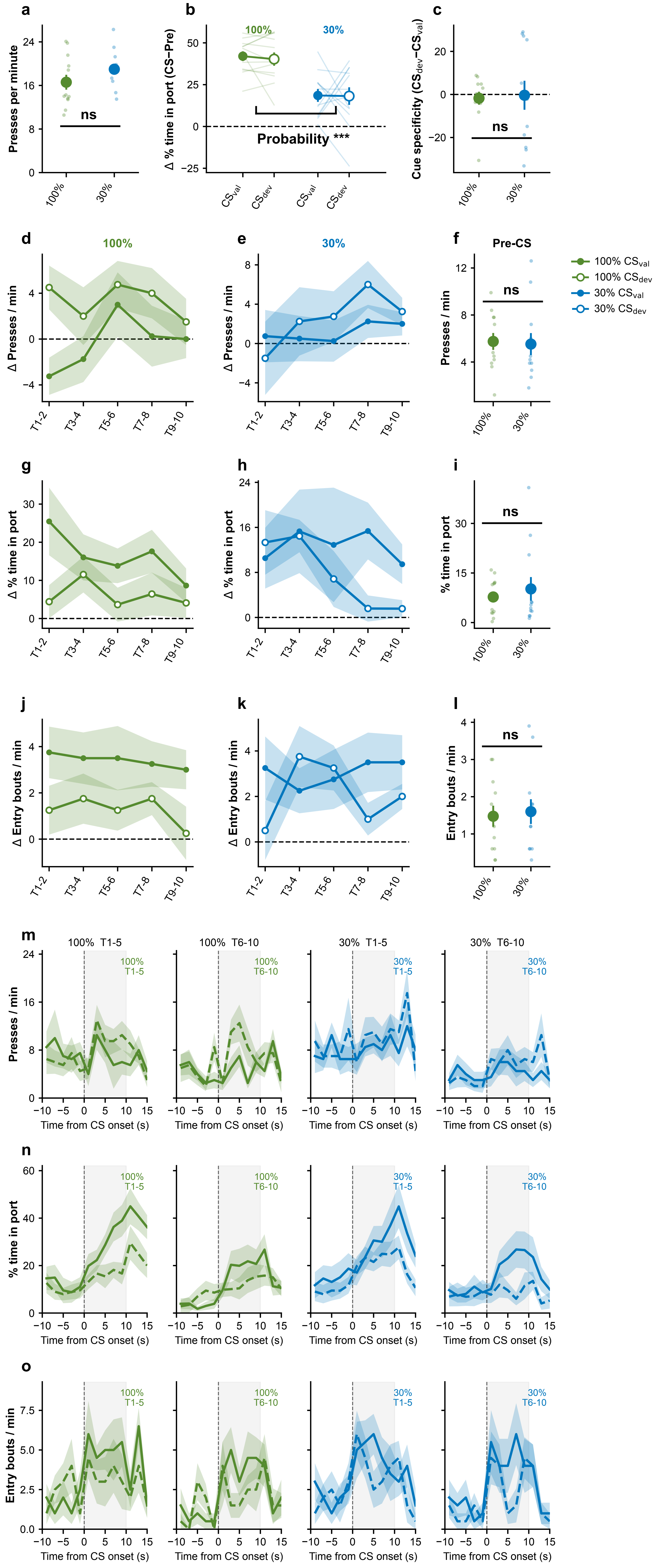

a
Presses per minute
ns
100%
30%
b
Δ % time in port (CS−Pre)
100%
30%
Probability ***
CSval
CSdev
CSval
CSdev
c
Cue specificity (CSdev−CSval)
ns
100%
30%
d
100%
Δ Presses / min
e
30%
Δ Presses / min
f
Pre-CS
Presses / min
ns
100% CSval
100% CSdev
30% CSval
30% CSdev
T1-2
T3-4
T5-6
T7-8
T9-10
g
Δ % time in port
h
Δ % time in port
i
% time in port
ns
j
Δ Entry bouts / min
k
Δ Entry bouts / min
l
Entry bouts / min
ns
m
100% T1-5
100% T6-10
30% T1-5
30% T6-10
Presses / min
Time from CS onset (s)
n
% time in port
o
Entry bouts / min
