# Supplementary material for: The Dorsomedial Prefrontal Cortex Uses Reward Predictions to Regulate How Rewards Are Pursued
Source: bioRxiv. 2026 Sep 3:2026.08.29.748037. Preprint. [Version 1] doi: 10.64898/2026.08.29.748037 (PMC13684574; doi:10.64898/2026.08.29.748037)
Supplement: Supplementary file 2 [file media-2.pdf]

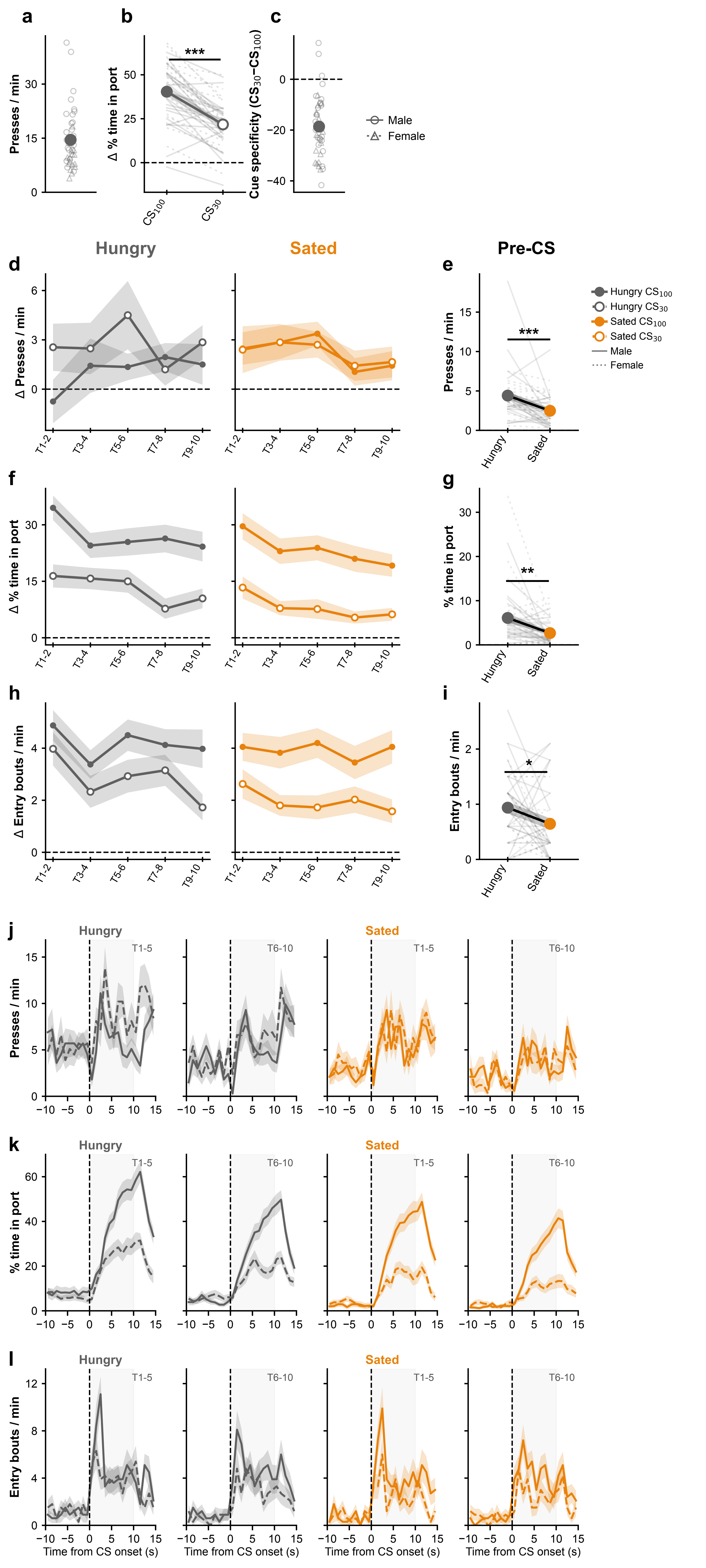

a
b
c
Presses / min
Δ % time in port
Cue specificity (CS30−CS100)
***
CS100
CS30
Male
Female
Hungry
Sated
Pre-CS
d
e
Δ Presses / min
T1-2
T3-4
T5-6
T7-8
T9-10
Hungry CS100
Hungry CS30
Sated CS100
Sated CS30
Male
Female
f
g
Δ % time in port
% time in port
**
h
i
Δ Entry bouts / min
Entry bouts / min
*
j
k
l
T1-5
T6-10
% time in port
Time from CS onset (s)
