# Supplementary material for: The Dorsomedial Prefrontal Cortex Uses Reward Predictions to Regulate How Rewards Are Pursued
Source: bioRxiv. 2026 Sep 3:2026.08.29.748037. Preprint. [Version 1] doi: 10.64898/2026.08.29.748037 (PMC13684574; doi:10.64898/2026.08.29.748037)
Supplement: Supplementary file 3 [file media-3.pdf]

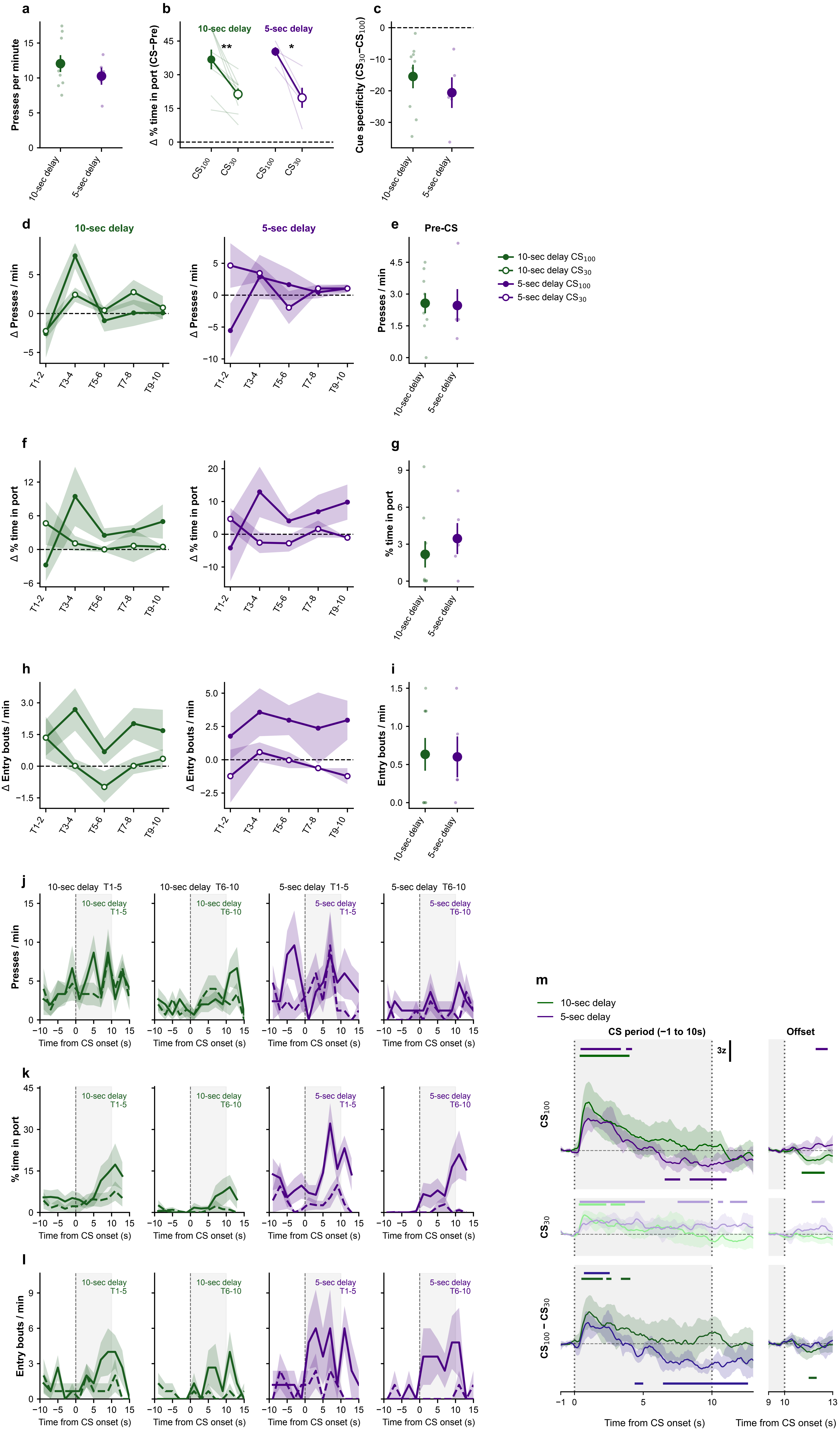
a
Presses per minute
10-sec delay
5-sec delay
b
10-sec delay
5-sec delay
Δ % time in port (CS−Pre)
**
*
CS_{100}
CS_{30}
c
Cue specificity (CS_{30}−CS_{100})
d
Δ Presses / min
T1-2
T3-4
T5-6
T7-8
T9-10
e
Pre-CS
Presses / min
10-sec delay CS_{100}
10-sec delay CS_{30}
5-sec delay CS_{100}
5-sec delay CS_{30}
f
Δ % time in port
g
% time in port
h
Δ Entry bouts / min
i
Entry bouts / min
j
10-sec delay T1-5
10-sec delay T6-10
5-sec delay T1-5
5-sec delay T6-10
Presses / min
Time from CS onset (s)
k
% time in port
l
Entry bouts / min
m
10-sec delay
5-sec delay
CS period (−1 to 10s)
Offset
3z
CS_{100}
CS_{30}
CS_{100} − CS_{30}
Time from CS onset (s)
