# Supplementary material for: The Dorsomedial Prefrontal Cortex Uses Reward Predictions to Regulate How Rewards Are Pursued
Source: bioRxiv. 2026 Sep 3:2026.08.29.748037. Preprint. [Version 1] doi: 10.64898/2026.08.29.748037 (PMC13684574; doi:10.64898/2026.08.29.748037)
Supplement: Supplementary file 4 [file media-4.pdf]

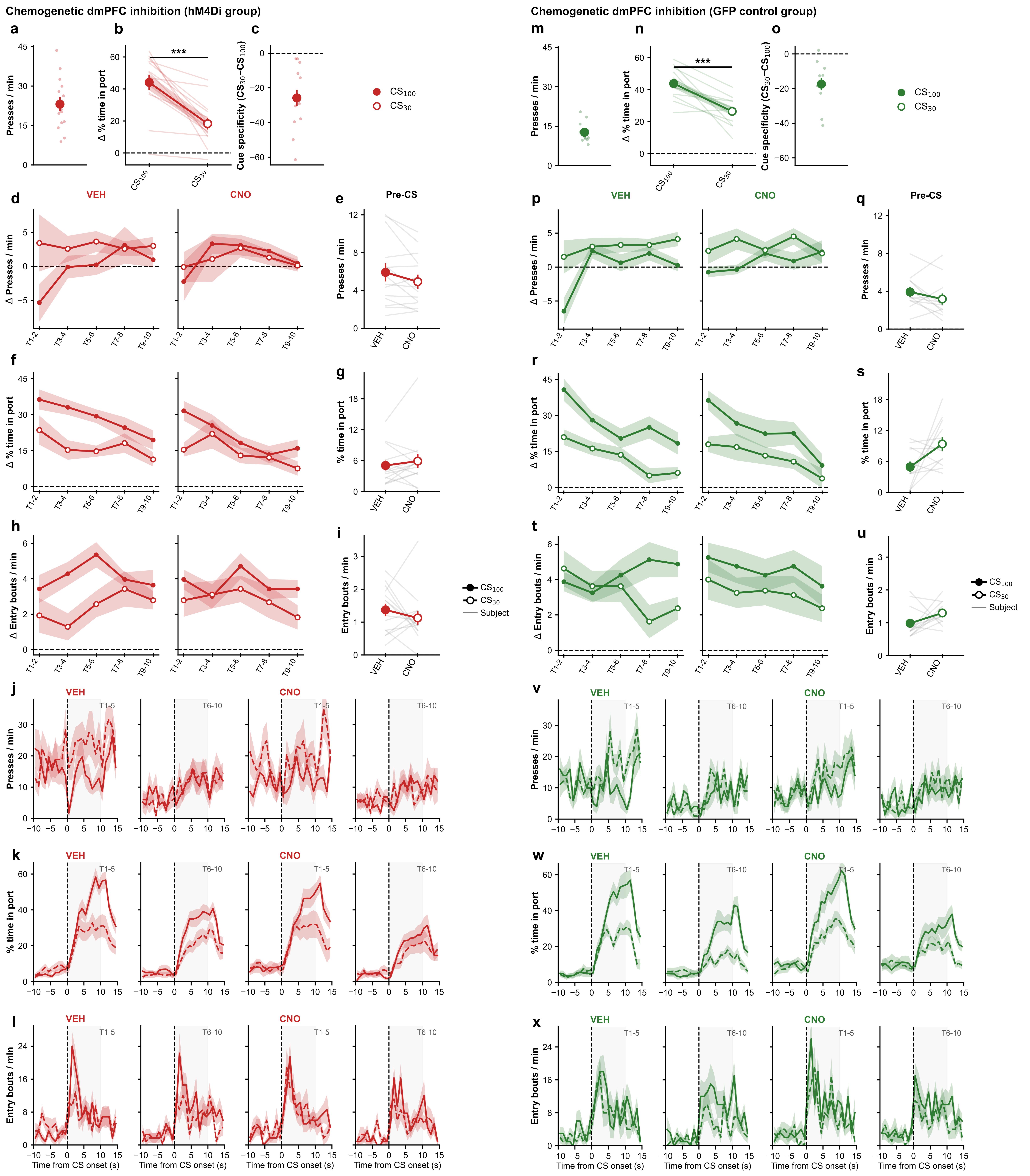
Chemogenetic dmPFC inhibition (hM4Di group)
Chemogenetic dmPFC inhibition (GFP control group)
a
b
c
m
n
o
Presses / min
Δ % time in port
Cue specificity (CS30−CS100)
***
CS100
CS30
d
VEH
CNO
e
Pre-CS
p
q
Δ Presses / min
T1-2
T3-4
T5-6
T7-8
T9-10
f
g
r
s
Δ % time in port
% time in port
h
i
t
u
Δ Entry bouts / min
Entry bouts / min
Subject
j
k
l
v
w
x
T1-5
T6-10
Time from CS onset (s)
