# Supplementary material for: The Dorsomedial Prefrontal Cortex Uses Reward Predictions to Regulate How Rewards Are Pursued
Source: bioRxiv. 2026 Sep 3:2026.08.29.748037. Preprint. [Version 1] doi: 10.64898/2026.08.29.748037 (PMC13684574; doi:10.64898/2026.08.29.748037)
Supplement: Supplementary file 5 [file media-5.pdf]

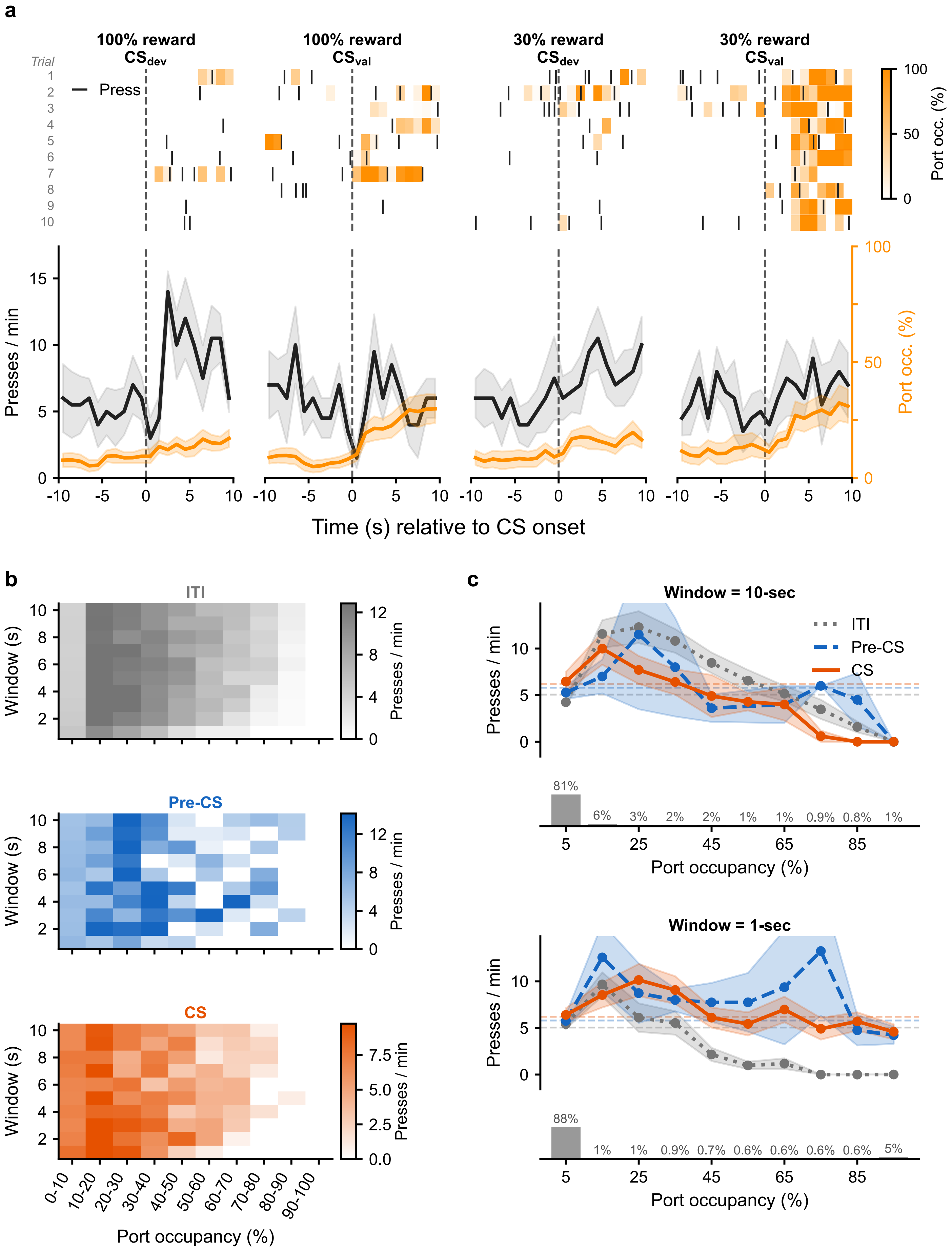

a
100% reward CSdev
100% reward CSval
30% reward CSdev
30% reward CSval
Trial
Press
Port occ. (%)
Presses / min
Time (s) relative to CS onset
b
ITI
Pre-CS
CS
Window (s)
Presses / min
Port occupancy (%)
c
Window = 10-sec
ITI
Pre-CS
CS
Presses / min
81%
6%
3%
2%
2%
1%
1%
0.9%
0.8%
1%
Port occupancy (%)
Window = 1-sec
88%
1%
1%
0.9%
0.7%
0.6%
0.6%
0.6%
0.6%
5%
Port occupancy (%)
