# Supplementary material for: The Dorsomedial Prefrontal Cortex Uses Reward Predictions to Regulate How Rewards Are Pursued
Source: bioRxiv. 2026 Sep 3:2026.08.29.748037. Preprint. [Version 1] doi: 10.64898/2026.08.29.748037 (PMC13684574; doi:10.64898/2026.08.29.748037)
Supplement: Supplementary file 6 [file media-6.pdf]

Main Tables

Table 1. Cue-specific regulation of instrumental pressing is intact under unperturbed conditions (pooled control-only sample).

n	Opportunity-adjustment	CS_{100} ΔPress rate (± SEM)	CS_{30} ΔPress rate (± SEM)	Effect ± SE	Test	p	Effect size [95% CI]
75	Unadjusted	0.28 ± 0.57	2.83 ± 0.76	2.54 ± 0.64	t(74) = 3.962	0.0002	*d* = 0.457 [0.218, 0.694]
75	Adjusted	2.65 ± 0.72	4.41 ± 0.80	1.76 ± 0.75	t(74) = 2.339	0.0220	*d* = 0.270 [0.039, 0.500]

Table 2. Reward devaluation releases cue-evoked pressing.

n	Term	Opportunity-adjustment	ΔPress rate Val ± SEM	ΔPress rate Dev ± SEM	Effect ± SE	Test	p	Effect size [95% CI]
24	Omnibus cue (devaluation effect, all groups)	Unadjusted			−2.55 ± 1.09	F(1,22) = 5.537	0.0280	η^2_p = 0.201 [0.000, 0.447]
24	Omnibus cue (devaluation effect, all groups)	Adjusted			−2.32 ± 1.29	F(1,22) = 3.168	0.0889	η^2_p = 0.126 [0.000, 0.374]
12	Val vs Dev @ 100% group	Unadjusted	−0.35 ± 0.80	3.35 ± 1.04	−3.70 ± 1.08	t(11) = −3.426	0.0057	d_z = −0.989 [−1.672, −0.277]
12	Val vs Dev @ 100% group	Adjusted	0.79 ± 0.96	4.12 ± 1.09	−3.33 ± 1.39	t(11) = −2.401	0.0352	d_z = −0.693 [−1.315, −0.047]
12	Val vs Dev @ 30% group	Unadjusted	1.15 ± 0.91	2.55 ± 1.36	−1.40 ± 1.88	t(11) = −0.745	0.4719	d_z = −0.215 [−0.783, 0.362]
12	Val vs Dev @ 30% group	Adjusted	2.43 ± 1.37	3.75 ± 1.43	−1.31 ± 2.21	t(11) = −0.594	0.5644	d_z = −0.172 [−0.738, 0.403]

Table 3. Satiety disrupts cue-specific regulation of instrumental pressing (State × Cue interaction).

n	Opportunity-adjustment	Test	p	Effect size [95% CI]
40	Unadjusted	F(1,39) = 5.442	0.0249	η^2_p = 0.122 [0.000, 0.315]
40	Adjusted	F(1,39) = 4.134	0.0489	η^2_p = 0.096 [0.000, 0.284]

Table 4. dmPFC inhibition disrupts cue-specific regulation of instrumental pressing (Drug × Cue interaction).

n	Group	Opportunity-adjustment	Test	p	Effect size [95% CI]
14	hM4Di	Unadjusted	$F(1,13) = 15.113$	0.0019	$\eta^2_p = 0.538$ [0.113, 0.725]
14	hM4Di	Adjusted	$F(1,13) = 9.113$	0.0099	$\eta^2_p = 0.412$ [0.031, 0.647]
12	GFP	Unadjusted	$F(1,11) = 1.040$	0.3297	$\eta^2_p = 0.086$ [0.000, 0.409]
12	GFP	Adjusted	$F(1,11) = 0.670$	0.4306	$\eta^2_p = 0.057$ [0.000, 0.374]

Opportunity adjustment expresses press rate per second of time not spent occupying the food port, so that cue-evoked differences in port occupancy cannot mechanically produce a difference in press rate. Unadjusted and adjusted analyses use different dependent variables and are both reported; they are not competing estimates of the same quantity. Adjusted press rate is computed separately for each subject and cue as a ratio of sums, dividing total presses by total time not spent in the food port across that cue's trials, and is applied to the CS and pre-CS periods alike (Figure 5).
